# Evidence of Adaptive Evolutionary Divergence during Biological Invasion

**DOI:** 10.1371/journal.pone.0049377

**Published:** 2012-11-12

**Authors:** Kay Lucek, Arjun Sivasundar, Ole Seehausen

**Affiliations:** 1 Aquatic Ecology and Evolution, Institute of Ecology & Evolution, University of Bern, Bern, Switzerland; 2 Department of Fish Ecology and Evolution, Centre for Ecology, Evolution and Biogeochemistry, EAWAG Swiss Federal Institute of Aquatic Science and Technology, Kastanienbaum, Switzerland; University of California, Berkeley, United States of America

## Abstract

Rapid phenotypic diversification during biological invasions can either arise by adaptation to alternative environments or by adaptive phenotypic plasticity. Where experimental evidence for adaptive plasticity is common, support for evolutionary diversification is rare. Here, we performed a controlled laboratory experiment using full-sib crosses between ecologically divergent threespine stickleback populations to test for a genetic basis of adaptation. Our populations are from two very different habitats, lake and stream, of a recently invaded range in Switzerland and differ in ecologically relevant morphological traits. We found that in a lake-like food treatment lake fish grow faster than stream fish, resembling the difference among wild type individuals. In contrast, in a stream-like food treatment individuals from both populations grow similarly. Our experimental data suggest that genetically determined diversification has occurred within less than 140 years after the arrival of stickleback in our studied region.

## Introduction

Numerous cases of rapid phenotypic diversification during biological invasions are known [1]–[3]. Many are thought to have arisen through adaptive phenotypic plasticity as a consequence of different selection pressures experienced during range expansion. Plasticity provides the possibility for rapid colonisation of new niches by expressing adapted phenotypes readily in different environments [2]–[4]. On the other hand, genetic divergence between populations based on alternative alleles of genes underlying ecologically relevant phenotypes can arise rapidly through natural divergent selection and such divergence can itself be enhanced by plasticity. However, few examples exist for evolutionary or adaptive diversification, defined here as divergence in heritable traits, in such evolutionarily young systems [1], [4], [5]. If phenotypic diversification emerges mainly through plasticity, diversification might be impeded between ecologically differentiated phenotypes, because selection can be dampened [3], [6]. In addition, the processes causing diversification during a biological invasion resemble the processes involved in adaptive radiations at an early stage [7], [8]. Hence, an identification of one of the abovementioned processes may shed light on the evolutionary pathways leading to apparently adaptive phenotypic diversification. Controlled laboratory experiments in which treatments differ in one or more key factors with all other conditions being the same, provide a powerful method to distinguish between genetically based divergence and plasticity in phenotypically differentiated populations [6], [9]–[11].

**Figure 1:**
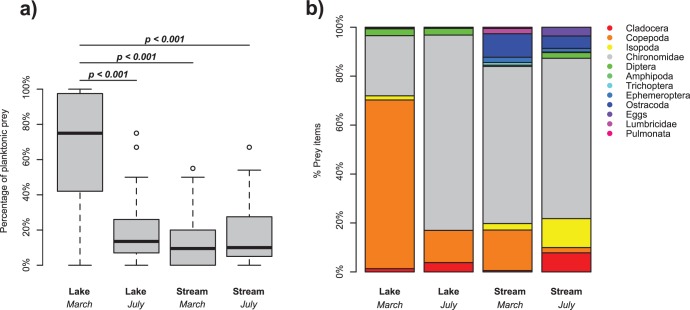
Summary of stomach content data from wild caught adults. a) Percentage of planktonic prey in the stomachs of adult stickleback caught either at the lakeshore or stream habitat before the beginning of the breeding season (March 2009) and during the breeding season (July 2007). Indicated significances are based on post hoc *t* tests for a generalized linear model among sampling events (see text for details). b) Relative abundance of prey items in the stomach of all fish pooled per sampling event.

A suitable candidate system for studying recent ecological diversification during biological invasion is the threespine stickleback (*Gasterosteus aculeatus*), in Switzerland. In its native range this fish species has repeatedly evolved divergently adapted freshwater ecotype pairs within the last 12,000 years. Many of the observed phenotypic shifts have been attributed to ancestral plasticity in the marine population [4], [12]. However, in some of these systems, indications for a genetic basis of adaptive diversification have been found [4], [6], [13]. These show fitness trade-offs between the differentiated coexisting sympatric ecotypes [6], [13], [14] and to a lesser degree in parapatric ecotypes [15]. In its invasive range in Lake Constance, Switzerland, ecologically distinct populations occur, living either in the lake or in streams and which differ in their trophic niches [1], [6], [9], [12], [13]. The stream dwelling populations feed mainly on benthic macroinvertebrates, whereas the lake dwelling population feeds mainly on zooplankton (Figure 1) and has longer gill rakers, suitable to filter small planktonic prey [1], [16]. Stable isotope data further supports ecological diversification into a mainly zooplankton feeding lake ecotype and a mainly benthos feeding stream ecotype (Sivasundar et al. *submitted*). This ecological diversification is striking as stickleback have only been introduced about 140 years ago in the Lake Constance region, deriving from a single East European genetic lineage as inferred from mitochondrial DNA [4], [17]. Neutral genetic markers further suggest genetic differentiation between the phenotypically divergent populations in this region [1], [4], [5], [16].

Here we test if the phenotypic and ecological differentiation that we observe in the invasive range of sticklebacks in Switzerland can be attributed to evolutionary divergence due to adaptation to different feeding regimes, which represents a major axis of divergence in our study system (Figure 1). Using a controlled laboratory experiment with full-sib F1 families, we test for differences in relative growth rates, measured as the overall difference in body size over time between a lake and a stream population when fed on either a “lake-like” (limnetic) or “stream-like” (benthic) diet. Evolutionary divergence is indicated if trait differences are maintained between the experimental groups. In addition, reduced growth would suggest adaptive differentiation if it is found in at least one population when fed on “foreign” food [2], [6], [11]. Alternatively if phenotypic diversification derives mainly from adaptive plasticity, individuals from both environments raised under identical conditions would express the phenotype that matches the laboratory rearing environment.

**Figure 2:**
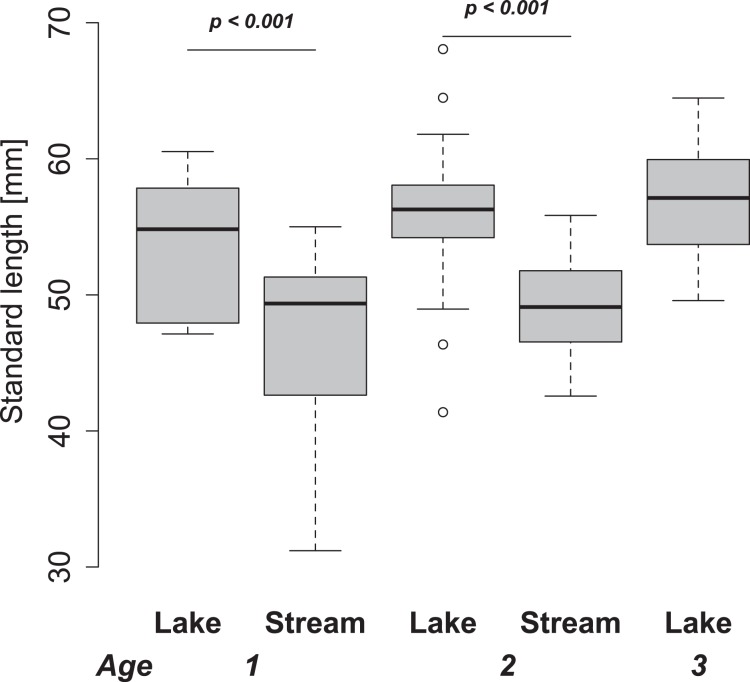
Distribution of standard lengths for the lake and stream habitat from wild caught adult individuals for each age class. No three-year-old stream fish were obtained. Significant size differences between habitats within an age class, based on Tukey’s HSD post hoc tests are indicated. Overall lake fish are significantly larger (*p<0.001*) when accounted for age.

Here, we use the increase in body size over time, which is related to the growth rate as a relative measure of fitness, since the wild populations studied here differ in their growth trajectory (Figure 2). This could reflect divergent adaptation due to e.g. different predation [7], [12] or feeding [6], [10], [18] regimes. We furthermore focused on body size as this trait can be easily estimated with little handling effort, which minimizes stress and reduced performance. We focus on the comparison of different ecotypes within an experimental feeding regime rather than comparing the regimes themselves because it provides a direct test for directional selection within each habitat, which may differ between habitats [11].

## Materials and Methods

### Pre-experimental Data Collection

In a preliminary study in July 2007 [4], wild adults from Lake Constance, Switzerland (47°29'02''N, 9°33'35''E) and from a stream, about 25 km upstream of the lake (47°19'33''N, 9°34'41''E) were sampled using minnow traps and by hand netting (N_Lake_ = 14, N_Stream_ = 32). Additional samples were obtained for both habitats in March 2009 (N_Lake_ = 25, N_Stream_ = 22). All fish were sacrificed in the field with an overdose of anaesthetic MS-222 and preserved in 95% ethanol for further analysis.

For each individual, stomachs were extracted and all food items were counted using a dissection microscope. Food items were assigned to the following taxonomic classes: *Amphipoda, Chironomidae, Cladocera, Copepoda, Diptera* imagos, *Ephemeropera, Isopoda, Lumbricidae, Ostracoda, Pulmonatae, Trichoptera,* and stickleback eggs. The percentage of planktonic prey was then calculated as the fraction of *Cladocera* and *Copepoda* to the total number of all food items present for each individual. Sampling events were statistically compared with a generalized linear model (GLM) assuming a quasibinomial distribution to account for over dispersion of the data. Pairwise significances were established using post hoc *t* tests. Two lake individuals from 2009 with empty stomachs were excluded. After extraction of the stomachs, all individuals were stained with formaldehyde and alizarin red to count their lateral plates for a different study (see [4], [8] for details).

### Experimental Fish Collection

Ripe individuals from the same sites as for the preliminary study were sampled in May 2010. Pairs (one male and one female) from the same source population were kept in individual 60×30×40 cm aquaria containing sand substrate, natural nesting material as well as a filtering and aerating system. After a successful spawning event the parental fish were removed. In addition to the individuals used for the crosses, a random subset of the wild population was preserved (N_Lake_ = 91, N_Stream_ = 49). These individuals were measured for their standard length. In addition, both otoliths, calcium carbonate structures in the inner ear that show seasonal rings, were extracted for each individual. Winter rings were counted at 40× magnification with a microscope to estimate the age of each individual. Age could not be determined for the individuals used in the preliminary study since the staining process dissolves calcium structures. Standard length was compared between habitats and age classes using an ANOVA with a Tukey’s HSD post hoc test. Overall differentiation was estimated with an ANOVA with age as a random factor to account for differences among age classes.

### Experimental Setup and Husbandry

Fertilized eggs were kept aerated in each tank. Eggs with fungal infection or dead embryos were removed daily. Two thirds of the water in each tank was replaced with well water every two days throughout the experiment. All hatched individuals were fed with *Artemia sp.* nauplii for the first five weeks after hatching. Between weeks four and five, small nematodes (*Panagrellus sp.*) were also provided. After this time, six stream families and seven lake families were randomly chosen. Each full-sib family was split into two subsets of 18–20 individuals each, one group being assigned to a "limnetic" type food regime, and the other to a "benthic" type food regime from week six onwards. The provided food items represent the main prey items eaten in the wild, based on the pre-experimental stomach content analyses (Figure 1). Consequently the treatments are referred to as “lake-like” for limnetic prey or “stream-like” for benthic prey. For the lake-like treatment, live zooplankton (mainly *Daphnia sp.* and limnetic copepods), collected from Lake Lucerne, Switzerland using a 170 µm zooplankton net, was provided every day. For the stream-like treatment, live bloodworms (*Chironomidae* spp. larvae) were provided daily. To require a more realistic benthic feeding behaviour from the fish, bloodworms were introduced through a plastic tube separating them from the fish and allowing them to attach to the substrate. The plastic tube was then removed after five minutes. Fish were fed once per day until week 23 after hatching. Individuals were not fed for 24 hours before the end of the experiment. After the experiment, all individuals were sacrificed with an overdose of anaesthetic MS-222, weighed to the nearest 0.01 g and preserved in 95% ethanol.

### Ethics

All necessary permits were obtained to sample sticklebacks for the described field studies from the St. Gallen cantonal fishery authorities. Fish husbandry followed the Swiss veterinary legislation in concordance with the federal veterinary office (FVO) and was approved by the cantonal office in Bern (Veterinärdienst des Kantons Bern).

### Estimating Growth through Time

**Figure 3:**
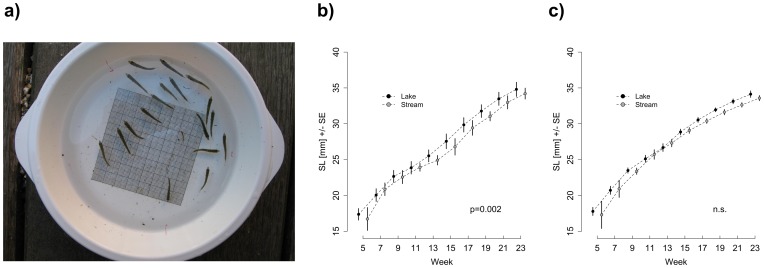
Summary of the increase in body size over time for experimental fish. (a) Illustration of the method used to estimate average family-based body size. A 1×1 mm grid was attached to the bottom of a standardized plastic container, where the water level was kept at 1.5 cm. Panels b and c show the average body size over time for lake and stream populations under either b) lake-like or c) stream-like food treatment. Dots represent the mean standard length (SL) of all families per source population (±1 SE).

Family-based differences in body size over time were estimated by taking standardised pictures of all individuals per tank in a plastic container with a 1×1 mm grid on the bottom and a water level of 1.5 cm (Figure 3a). Pictures were taken every two weeks starting on the first treatment day. Standard length of each individual was measured using ImageJ 1.43i [8], [14], [16] using the grid on each picture as a reference. Individual size at the beginning of the experiment was compared between source populations and treatments using a linear mixed effect model with family as random factor. Relative growth rates, measured as the difference in size over time, were statistically compared between source populations within treatments using a repeated measurement ANOVA with families as random factor. Experimental week was treated as a numerical variable, which allowed the estimation of the overall trend over time. Comparisons across treatments were not performed except for the comparison at the beginning of the experiment up to which point all individuals should have experienced a similar raising environment (i.e. *Artemia* nauplii and *Panagrellus*). All statistical analyses were performed in R 2.12.1 (The R Core Team 2010).

## Results

### Differentiation of Wild Fish

The percentage of planktonic prey found in stomachs differed significantly across sampling events (*Χ*_3_ = 19.20, *p*<0.001), being significantly higher in the lake population, sampled in March 2009 compared to both stream samplings (March: *t = *−4.09, *p*<0.001; July: *t* = −8.99, *p*<0.001) and the lake population sampled in July (*t* = −7.50, *p*<0.001) (Figure 1a). However, the lake population sampled in July did not differ in the percentage of planktonic prey from the stream populations sampled in March (*t* = 0.02, *p* = 0.987) or July (*t* = −1.57, *p* = 0.121). Wild caught lake fish fed mainly on cladocerans in March with a relatively small fraction of chironomid larvae, whereas the stream fish feed mainly on chironomids (Figure 1b). In July individuals from both habitats fed predominantly on chironomids.

The wild caught fish that were obtained together with the parents of the experimental individuals differed significantly in age between habitats, with lake fish being older than stream fish (average lake: 2.4 years, average stream: 1.7 years; *F*_1,138_ = 44.07, *p*<0.001). Size differed consistently between habitats for one and two year old individuals with lake fish being consistently larger (Figure 2), whereas size did not statistically differ between age classes within habitat (all *p*>0.05). Overall, lake fish were significantly larger than stream fish when age was accounted for (*F*_1,137_ = 57.45, *p*<0.001).

### Experimental Fish

In total, 441 out of 511 individuals survived until the end of the experiment (average overall mortality: 13.9%±16.1% SD). Mortality was highest for lake fish in the lake-like treatment (24.0%±26.1% SD) and lowest for stream fish in the lake-like treatment (4.1%±3.3% SD), whereas mortality was relatively similar in the stream-like treatment (lake fish: 12.6%±9.2% SD; stream fish: 14.2%±9.2% SD). Mortality was however not statistically different between treatments (*F*_1,22_ = 0.04, *p* = 0.849) or source populations (*F*_1,22_ = 2.54, *p* = 0.126) with a non significant interaction (*F*_1,22_ = 3.46, *p* = 0.076) between them.

Although individuals were randomly assigned to each treatment, standard length differed between treatment groups five weeks after hatching at the beginning of the experiment, with individuals in the stream-like treatment being significantly larger (*F*_1,495_ = 18.85, *p*<0.001). Source populations on the other hand did not differ (*F*_1,11_ = 0.10, *p* = 0.754), and the interaction of source and treatment was not significant (*F*_1,495_ = 0.36, *p* = 0.548).

Size differed significantly over time between the lake and the stream population in the lake-like treatment (*F*_1,2382_ = 9.66, *p* = 0.002) with lake fish growing larger than stream fish (Figure 3b). In the stream-like treatment, populations did not differ significantly in body size over time (*F*_1,2299_ = 2.03, *p* = 0.155, Figure 3c). For both stream and lake populations, individuals in the lake-like treatment grew faster than those in the stream-like treatment (stream: *F*_2,2235_ = 10.44, *p* = 0.001; lake: *F*_2,2457_ = 10.97, *p*<0.001). At the end of the experiment, fish from the lake-like treatments (regardless of source population) were slightly longer (*F*_1,427_ = 12.06, *p*<0.001), but did not differ in body weight (*F*_1,427_ = 0.05, *p* = 0.810) compared to fish from the stream-like treatments. Experimental fish did not differ between source populations at the end of the experiment (length: *F*_1,11_ = 0.84, *p* = 0.381; weight: *F*_1,11_ = 2.02, *p* = 0.183) with the interaction between source population and treatment being not significant for both length (*F*_1,426_ = 0.39, *p* = 0.531) and weight (*F*_1,426_ = 0.36, *p* = 0.549).

## Discussion

In this study, we experimentally tested for a genetically determined evolutionary diversification during a biological invasion in a species known to occasionally form ecotype pairs within its natural range [6], [6], [9], [12], [13,15]. We find that in the lake-like food treatment lake fish grow faster than stream fish. In the stream-like food treatment on the other hand, we find no significant difference between individuals from the two populations in their growth. These results provide experimental indications for putatively adaptive diversification, associated with the exploitation of different ecological niches can occur during a biological invasion. This has otherwise been shown only in few cases [12], [16], [19], [20], where adaptation and diversification have mostly been only indirectly inferred (e.g. [17], [21], [22]). However, phenotypic diversification in newly colonised habitats is a common phenomenon in invasive species [5], [12], [16], [19], [20]. Given that it provides the possibility to express advantageous phenotypes readily in a broad range of environments, phenotypic plasticity has often been invoked to explain phenotypic divergence in general [2], [6], [9], [12], [13] and for stickleback in particular [12,12]. In contrast, we found indications for a genetically determined fitness component separating the two ecotypes after less than 140 years since introduction in one comparison. Such a genetic basis could derive from multiple introduction events where different genetic lineages could admix. This could then lead to an increase of the adaptive genetic potential in the admixed population upon which divergent selection can act [18]. Alternatively *in situ* evolution potentially based on ancestral standing genetic variation may account for the observed diversification. Because both populations originate from the same genetic lineage [4], diversification has likely occurred *in situ*. However, we are not able to determine if the lake population evolved from the stream population or *vice versa* through divergent adaptation. The first scenario seems to be more likely as sticklebacks were historically first observed in a stream close to our stream site in 1870 [4].

Niche expansion during invasion, i.e. the colonisation of divergent habitats, together with an increase in the diversity of utilised resources, may be attributed to ecologically driven diversification. This could represent a first step towards adaptive diversification [8], where heritable specialisation characterizes the second step along the invasion-diversification continuum [8], [16]. Fitness trade offs between populations may arise if ecotypic specialisation for different resources occurs as a result of divergent natural selection [6]. Further selection could then lead to the fixation of alternative phenotypes with their underlying genotypes between ecologically differentiated populations, ultimately leading to ecological speciation [23]. Similarly, rapid phenotypic differentiation and diversification in sticklebacks, especially in body shape and defense related phenotypes has been shown to occur repeatedly along the marine – freshwater transition [12], [19], [20]. Here, the rapid differentiation in lateral plate number has been attributed to selection on standing genetic variation [21], [22]. Experimental assessments for a genetic differentiation in feeding related phenotypic traits have however only rarely been conducted, which suggest a mainly plastic contribution [12], [19], [20].

Our finding that lake fish are able to utilise limnetic prey better than stream fish compared to benthic prey where lake fish grow at similar rates as stream fish indirectly suggests adaptive diversification along a parapatric benthic-limnetic axis. This is consistent with ecotype formation of sticklebacks in their natural range, where similar ecotypes as the ones observed here usually evolved over millennia and where divergence therefore is likely to be much older than in our study system [6], [9], [12], [13], [15]. In these systems, consistent adaptive divergence was found for both sympatric ecotypes [6], [13], whereas a reciprocal transplant experiment between parapatric lake and stream populations showed different responses in each tested environment [15]. In the later case, lake dwelling fish grew faster than stream dwelling ones in a lake environment, whereas both grew similarly in the stream environment. Using a controlled laboratory experiment, we obtain a similar pattern. The difference between these sympatric and parapatric comparisons may arise through different strengths of divergent selection, i.e. where intraspecific competition may increase divergent selection or cause disruptive selection in sympatry but not in parapatry [11].

In concordance with the abovementioned experiments in the wild, our experiment suggests that lake fish are able to grow on stream-like food at the same rate as stream fish, and may intrinsically grow faster and bigger. Although size differed only marginally between source populations within one of our experimental treatments at any point in time, the repeated measurement ANOVA supports a significant difference in body size through time in the lake-like treatment feeding on limnetic prey, suggesting a different growth rate between the ecotypes. This observed growth difference could result in different adult sizes, which is consistent with the size differences observed in the wild, where lake fish are significantly larger even when corrected for their age (Figure 2).

The absence of differentiation in the stream-like treatment could may further suggest different levels of local adaptation between the two tested populations [11]. Therefore behavioural versatility may be maintained, which would allow lake fish to switch more readily between the different feeding regimes [12]. Such behavioural versatility can be beneficial in heterogeneous environments where individuals encounter different feeding regimes. This may be the case in our system where lake fish feed on plankton in the open lake outside the breeding season, but enter shallow inshore waters, such as stream mouths for breeding. Here they start to increase feeding on benthic food, which is the most common locally available prey type. Stream fish on the other hand forage in streams throughout the year where they predominantly feed on locally available benthic prey (Figure 1). Consequently specialization may be reduced in the lake population as a consequence of the more heterogeneous feeding environment in comparison to the stream population, which feeds predominantly on benthic macroinvertebrates throughout the year. However, our experimental setup using F1 offspring from wild parents does not allow to exclude potential maternal effects, which could be responsible for the higher intrinsic growth rate in stream fish. Indeed the parental populations used in our experiment differed in both their average age and their body size, which suggests a different life history strategy [24]. Such maternal effects could be either environmentally or genetically determined and hence be adaptive as well [24], but further experiments are needed to estimate the contributions of maternal effects.

The observed pattern between the wild populations, where size differs between habitats in all age classes but age classes do not differ within habitats suggests that divergence in growth rates occurs mainly during the first year of life. Such size difference could be caused by selection due to different predation pressures in the two habitats, since increased body size could facilitate escape from gape-limited predators [11], [20], [16]. Indeed, experimental evidence suggests that sticklebacks are able to increase their growth rate as a plastic response to the presence of a predator, where larger individuals escape gape limited predators [2], [3], [7]. In contrast, our experimental individuals were not exposed to predators, suggesting a genetic basis for increased growth rather than plasticity. Furthermore, even if predation is a main driver for the observed divergence in growth rates in the wild, further adaptations are needed to feed on zooplankton, i.e. forming limnetic feeding type phenotypes in sticklebacks [2], [3], [12]. Although divergence in specific feeding related phenotypes has been shown before in our system [1], [5] especially in gill raker length (Sivasundar et al. *submitted*), our experiment did not allow to investigate the relative growth trajectories of these traits as they either require increased handling or the individual to be sacrificed (e.g. gill rakers) [3], [25].

The evolutionary diversification that we observe in our study system may have further implications for both the present native species and the ecosystem itself. By exploiting different niches, sticklebacks are likely to introduce divergent selection pressure through interspecific competition and divergent predation pressure on their prey [5], [8]. Moreover, it has been shown that in their native range divergent stickleback ecotypes can each affect the community composition of lower trophic levels in different ways [6], [9], [26]. This can further change the trophic interactions of other species. Indeed, experimental evidence suggests that lake dwelling sticklebacks from Lake Constance exert strong predation pressure on important herbivorous macroinvertebrates [12], [27]. Here, stickleback predation changes both population size and growth of the prey by altering the sex ratios of the herbivores, which could then affect the ecosystem by increasing the vegetation density [6], [13], [27]. Consequently, invasive sticklebacks [4], [6], [13], [28] might serve as a model system to further study evolutionary aspects and consequences of species invasion.
